# Dietary Chloride Deficiency Syndrome: Pathophysiology, History, and Systematic Literature Review

**DOI:** 10.3390/nu12113436

**Published:** 2020-11-09

**Authors:** Giulia C. Signorelli, Mario G. Bianchetti, Luca M. M. Jermini, Carlo Agostoni, Gregorio P. Milani, Giacomo D. Simonetti, Sebastiano A. G. Lava

**Affiliations:** 1 Facoltà di Scienze Biomediche, Università della Svizzera Italiana, 6900 Lugano, Switzerland; GiuliaCarlotta.Signorelli@gmail.com (G.C.S.); mario.bianchetti@usi.ch (M.G.B.); jermini.luca@bluewin.ch (L.M.M.J.); giacomo.simonetti@eoc.ch (G.D.S.); 2 Pediatric Institute of Southern Switzerland, Ospedale San Giovanni, 6500 Bellinzona, Switzerland; 3 Pediatric Unit, Fondazione IRCCS Ca’ Granda Ospedale Maggiore Policlinico, 20122 Milan, Italy; carlo.agostoni@unimi.it; 4 Department of Clinical Sciences and Community Health, Università degli Studi di Milano, 20122 Milan, Italy; 5 Italian Society for Pediatric Gastroenterology Hepatology and Nutrition (SIGENP), 20126 Milan, Italy; 6 Pediatric Cardiology Unit, Department of Pediatrics, Centre Hospitalier Universitaire Vaudois, and University of Lausanne, 1011 Lausanne, Switzerland; webmaster@sebastianolava.ch

**Keywords:** chloride, formula milk, acid–base balance

## Abstract

Metabolic alkalosis may develop as a consequence of urinary chloride (and sodium) wasting, excessive loss of salt in the sweat, or intestinal chloride wasting, among other causes. There is also a likely underrecognized association between poor salt intake and the mentioned electrolyte and acid–base abnormality. In patients with excessive loss of salt in the sweat or poor salt intake, the maintenance of metabolic alkalosis is crucially modulated by the chloride–bicarbonate exchanger pendrin located on the renal tubular membrane of type B intercalated cells. In the late 1970s, recommendations were made to decrease the salt content of foods as part of an effort to minimize the tendency towards systemic hypertension. Hence, the baby food industry decided to remove added salt from formula milk. Some weeks later, approximately 200 infants (fed exclusively with formula milks with a chloride content of only 2–4 mmol/L), were admitted with failure to thrive, constipation, food refusal, muscular weakness, and delayed psychomotor development. The laboratory work-up disclosed metabolic alkalosis, hypokalemia, hypochloremia, and a reduced urinary chloride excretion. In all cases, both the clinical and the laboratory features remitted in ≤7 days when the infants were fed on formula milk with a normal chloride content. Since 1982, 13 further publications reported additional cases of dietary chloride depletion. It is therefore concluded that the dietary intake of chloride, which was previously considered a “mendicant” ion, plays a crucial role in acid–base and salt balance.

## 1. Introduction

Approximately 40 years ago, some infants were found to suffer from failure to thrive, constipation, food refusal, muscular weakness, and delayed psychomotor development associated with metabolic alkalosis. The diagnostic work-up disclosed a reduced urinary chloride excretion. Notably, these infants were fed exclusively with formula milks that had been marketed shortly before. Finally, further investigations established that the chloride level was very low in the new formula milks. Hence, the existence of a new peculiar form of chloride deficiency metabolic alkalosis was suspected and subsequently confirmed [1].

The aims of this communication are to concisely describe the pathophysiology of dietary chloride-depletion metabolic alkalosis, and to systematically address the clinical literature on this disturbance.

## 2. Chloride Deficiency Metabolic Alkalosis

Metabolic alkalosis is a common acid–base derangement. It occurs when there is an accumulation of base or a net loss of acid from the extracellular fluid, and is recognized by an increase in blood bicarbonate (HCO_3_^−^) and pH. The increase in blood pH depresses ventilation and results in elevated partial pressure of carbon dioxide (pCO_2_). The partial pressure of carbon dioxide increases by about 0.5 to 0.7 mm Hg for every 1.0 mmol/L increase in blood bicarbonate [2].

Chloride deficiency (often but not always caused by extracellular fluid volume depletion) plays a fundamental causative role in metabolic alkalosis. The development, and subsequently the maintenance, of metabolic alkalosis generally have two different explanations. (a) Development: an elevated bicarbonate concentration is due to excessive intestinal or urinary hydrogen loss; hydrogen ion movement into cells; administration of anions such as citrate, lactate, or bicarbonate; and volume contraction around a relatively constant amount of extracellular bicarbonate. (b) Maintenance: an elevated bicarbonate concentration is maintained because of an inability to eliminate the excess bicarbonate in the urine [2,3].

**Figure 1:**
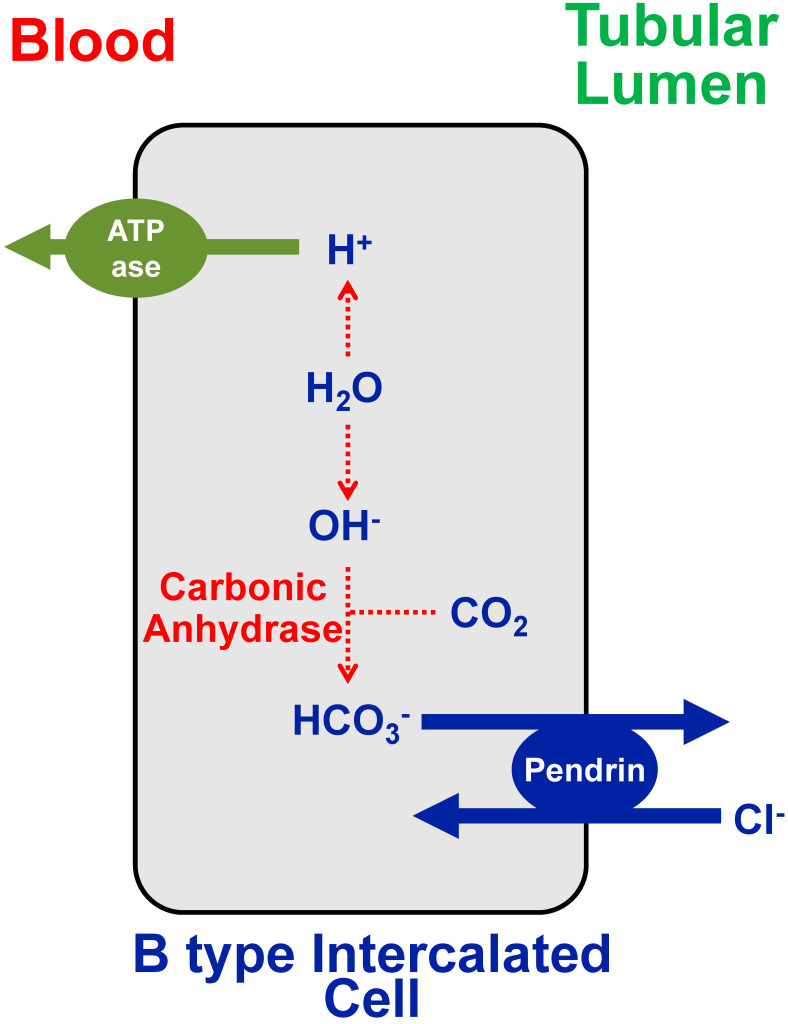
Sketch depicting a B type intercalated cell (also termed ß-intercalated cells) in the kidney cortical collecting duct and the connecting tubule. This cell expresses the Cl^−^/HCO_3_^−^ exchanger pendrin on the tubular plasma membrane, and the H^+^-ATPase (= H^+^-pump) on the basolateral (“blood”) plasma membrane. Metabolic acidosis downregulates pendrin expression and therefore tends to reduce bicarbonate secretion. On the other hand, metabolic alkalosis upregulates pendrin and therefore tends to secrete bicarbonate into the tubular lumen.

The chloride–bicarbonate (Cl^−^/HCO_3_^−^) exchanger pendrin, which is located on the tubular membrane of B type intercalated cells (also termed ß-intercalated cells) in the kidney cortical collecting duct and the connecting tubule (as shown in Figure 1), is sodium-independent and secretes bicarbonate into the lumen in exchange for the reabsorption of chloride [4,5]. It plays a major role, among others, in acid–base balance, as indicated by the fact that it is downregulated in acidosis and upregulated following bicarbonate loading (e.g., elevation in blood bicarbonate concentration, as seen with an alkaline diet (i.e., rich in fruits and vegetables)).

**Figure 2:**
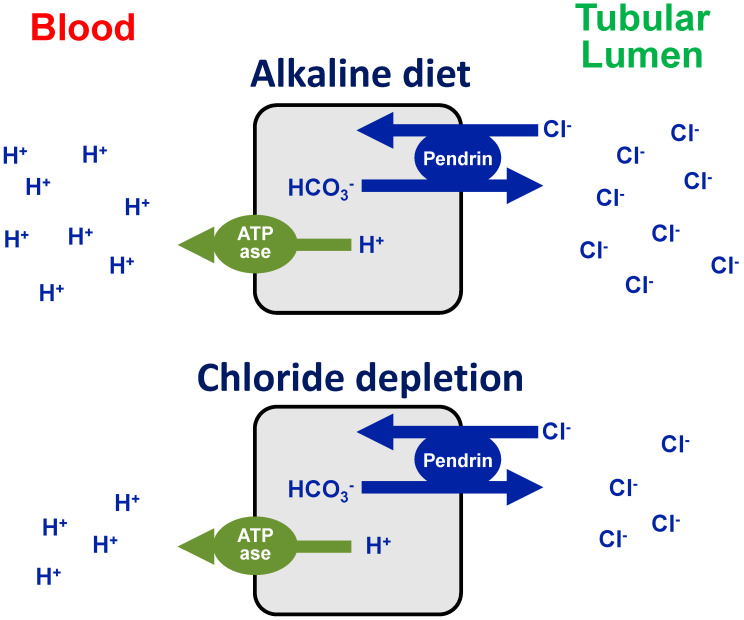
Sketch depicting the corrective response of the sodium-independent chloride–bicarbonate exchanger pendrin to metabolic alkalosis induced by an alkaline diet (upper panel), or by dietary chloride deficiency (lower panel). In order for pendrin to secrete bicarbonate and, therefore, correct alkalosis, chloride must be reabsorbed. Thus, sufficient distal tubule chloride delivery is critical for bicarbonate secretion (as in alkaline diet, upper panel). However, chloride depletion (and reduced effective blood volume) decreases distal chloride delivery, and this reduces pendrin-mediated bicarbonate secretion (and chloride reabsorption), thereby “maintaining” metabolic alkalosis (lower panel).

In order for pendrin to secrete bicarbonate, chloride must be reabsorbed. Thus, sufficient distal tubule chloride delivery is critical for bicarbonate secretion. However, chloride depletion decreases distal chloride delivery, and this reduces pendrin-mediated bicarbonate secretion (and chloride reabsorption), thereby “maintaining” metabolic alkalosis (Figure 2). The main causes of chloride deficiency metabolic alkalosis [2,3], which include urinary salt wasting, excessive loss of salt in the sweat, intestinal chloride wasting, and poor dietary chloride intake, are depicted in Table 1.

## 3. Epidemic and Sporadic Dietary Chloride Depletion Alkalosis: A Systematic Review

### 3.1. Literature Search Strategy

This review was accomplished in accordance with the Preferred Reporting Items for Systematic Reviews and Meta-Analyses recommendations [6]. Computerized searches were run in the databases of the United States National Library of Medicine, Excerpta Medica, and Web of Science on 31 March and repeated on 31 July 2020. Original reports and letters published with no language limits were considered. The search strategy incorporated the terms “dietary chloride deficiency” OR “dietary chloride depletion” OR “chloride deficiency” OR (“chloride depletion” OR hypochloremia AND diet). References listed within bibliographies of the retrieved records and articles already known to the authors were also considered for inclusion.

Two authors (GCS and MGB) autonomously screened all identified titles and abstracts in a non-blinded fashion. Upon retrieval of candidate reports, full-text publications were reviewed for eligibility.

### 3.2. Data Extraction

The following data were retrieved from the selected studies: authors, year of publication, origin and type of population, laboratory data, type of diet, clinical manifestation, diagnosis, follow-up, and clinical outcome. Two researchers (GCS and MGB) independently performed data extraction and inserted relevant information in a predefined database. Discrepancies in data extraction were resolved by consensus.

### 3.3. Search Results

**Figure 3:**
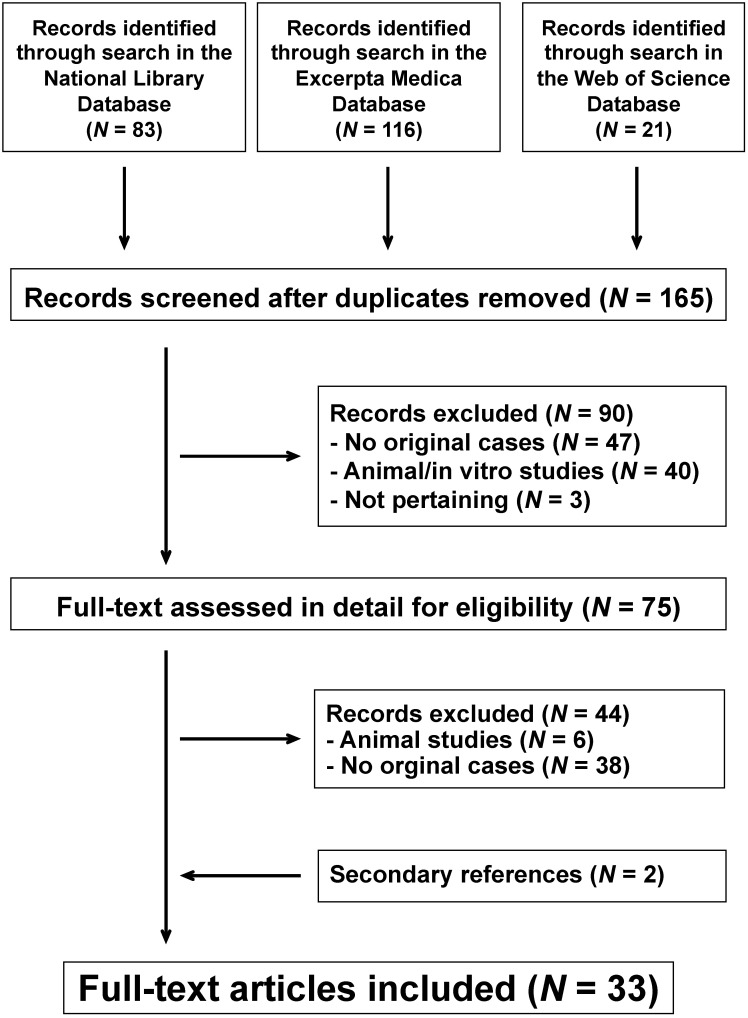
Dietary chloride deficiency syndrome. Flowchart of the literature search process.

The literature search process is summarized in Figure 3. For the final analysis, we retained 33 reports published since 1979. They had been reported from the following countries: United States of America (*N* = 21), Italy (*N* = 2), Spain (*N* = 2), Canada (*N* = 1), France (*N* = 1), Great Britain (*N* = 1), Greece (*N* = 1), Japan (*N* = 1), the Netherlands (*N* = 1), South Africa (*N* = 1), and South Korea (*N* = 1).

### 3.4. Epidemic Dietary Chloride Depletion Alkalosis in Infants Fed a Low-Chloride Formula Milk

#### 3.4.1. Historical Background

In the late 1970s, recommendations were made to decrease the salt content of infant foods as part of an effort to minimize the development of systemic hypertension in adulthood. The Committee on Nutrition of the American Academy of Pediatrics established suggested minimum concentrations of electrolytes for infant formulas: the recommendations included a minimum of 11 mmol/L of chloride, nearly equivalent to the concentration in human milk. In 1977, the baby food industry decided to remove added salt from formula milk [1,7].

#### 3.4.2. Outbreaks in the United States of America (1979) and Spain (1981)

In June 1979, Dr. Shane Roy (21 July 1935–28 March 2009), a pediatric hospitalist in Memphis, Tennessee, examined a 6-month-old girl who had been admitted with failure to thrive, constipation, food refusal, muscular weakness, and delayed psychomotor development [8]. In this infant, whose blood pressure was normal, the laboratory work-up disclosed severe hypokalemia and metabolic alkalosis. Very soon, five further cases were presented to S. Roy [9]. A total of approximately 150 similar cases from the United States and Puerto Rico were subsequently reported [10,11,12,13,14,15,16,17,18].

In August 1981, a 4-month-old boy with the aforementioned clinical and biochemical features was presented to Dr. Juan Rodríguez-Soriano (5 March 1933–13 October 2010) in Bilbao, Spain. This prominent pediatric kidney disease specialist subsequently identified and reported 29 additional cases [19].

The laboratory features observed in the babies living in both America and Spain, whose ages ranged from 0.5 to 10, with a median age of 5 months, are summarized in Table 2. In addition to metabolic alkalosis, hypokalemia, hypochloremia, reduced urinary chloride excretion (in most cases urinary chloride was undetectable), slightly altered renal function parameters (with an increased urea-to-creatinine ratio), and an activated renin–angiotensin–aldosterone system were noted. Hypercalcemia, hyperphosphatemia, hypercalciuria, and hypermagnesuria were disclosed in many Spanish infants [18]. Microhematuria was also detected in many cases [9,19]. Hence, the existence of a potential for nephrocalcinosis was suggested. However, no imaging studies were performed.

A renal biopsy, performed in two American infants, did not disclose evidence for juxtaglomerular hyperplasia [9]. Some tubular calcification was noted in one of the two cases.

All these infants were found to be fed exclusively with three modified formula milks that had been marketed shortly before. The babies living in the United States had been fed two soy-based formula milks [8,18] named Neo-Mull-Soy^®^ (Syntex Labs, Palo Alto, CA, USA) and CHO-Free^®^ (Syntex Labs, Palo Alto, CA, USA). On the other hand, the 30 babies living in Spain [19] had ingested the cow-milk formula Aptamil-1^®^ (Milupa SA, Alicante, Spain).

Laboratory studies found that [1,8,9,18,19], contrary to product information, the chloride levels of Neo-Mull-Soy^®^, CHO-Free^®^, and Aptamil-1^®^ were only 2–4 mmol/L, substantially below the 11 mmol/L recommended by the authorities. As a consequence, these infants had a chloride intake of 0.3–0.6 mmol/kg daily, as opposed to the recommended daily intake of 3–6 mmol/kg.

In both North American and Spanish infants, failure to thrive, constipation, food refusal, muscular weakness, and the altered laboratory values remitted within ≤7 days on formula milk with a normal chloride content (salt supplementation was initially given to some of them).

The companies voluntarily stopped manufacturing the formulas Neo-Mull-Soy^®^, CHO-Free^®^, and Aptamil-1^®^; halted the distribution to wholesalers; and requested that wholesalers stop sales to retailers. Furthermore, they notified healthcare professionals about the problem. It has subsequently been estimated that a total of approximately 50,000 infants had been exposed to these defective formulas [1].

### 3.5. Dietary Chloride Depletion Alkalosis after the Initial (1979–1981) Outbreaks

After 1982, 13 reports addressed the occurrence of dietary chloride depletion.

In five breastfed boys aged from 7 weeks to 10 months (median 6 months), a tendency to metabolic alkalosis, hypokalemia, and reduced urinary chloride excretion was noted [20,21,22]. In these infants, history, clinical findings, and laboratory features were inconsistent with a diagnosis of persistent vomiting, congenital chloride diarrhea, diuretic use, and cystic fibrosis. Hence, the diagnosis of dietary chloride depletion alkalosis was suspected and subsequently confirmed based on very low chloride levels in the mother’s milk, and the resolution of symptoms and biochemical abnormalities occurred once the infant’s nutrition was changed to a commercial formula preparation. The dietary habits of the mothers were not addressed in these studies.

Nine further cases were observed in non-breastfed infants (six boys and three girls) aged from 4 weeks to 14 months (with a median age of 6 months). After excluding the causes of chloride deficiency discussed for the five aforementioned breastfed infants, the possible diagnosis of dietary chloride deficiency was suspected [23,24,25,26,27,28,29]. Meticulous history-taking revealed that the children were on unconventional formulas: hypoallergenic formulas (*N* = 3), almond milk (*N* = 3), rice milk (*N* = 2), and homemade blended formula (*N* = 1). The chloride concentration in milk, assessed exclusively in one of the mentioned cases [27], was found to be very low. In these cases, clinical symptoms and laboratory abnormalities normalized once the feeds were replaced by a commercial formula preparation.

Fifty-nine Japanese patients with severe motor and intellectual disability (15 children ≤20 years of age, and 44 adults) were on tube feeding [30]. Approximately 2 months after changing to two new liquid nutritional products, weight loss and a tendency to mild bowel movement disorders including constipation or diarrhea were noted, which were associated with hypochloremia (*N* = 30), hypokalemia (*N* = 26), and hyponatremia (*N* = 20). Furthermore, the chloride level of the nutritional product was found to be ≤10 mmol/L. The patients’ symptoms and laboratory abnormalities were resolved after supplementation with sodium chloride (with or without potassium chloride). Furthermore, the new liquid nutritional products were replaced with the old one [30].

Finally, the combination of endurance exercise and a diet low in salt and protein and high in vegetables may cause chloride deficiency hypokalemic metabolic alkalosis [31,32].

## 4. Possible Long-Term Sequelae after Exposure to a Low-Chloride Formula Milk

### 4.1. Cognitive Deficits

It has been speculated that malnutrition in early infancy, including among others a brief period of starvation, may result in poor learning abilities [1]. Following the 1979 outbreak of dietary chloride deficiency, the Centers for Disease Control established a registry of children who had been exposed to a chloride-deficient formula with or without evidence of hypochloremic metabolic alkalosis.

Hellerstein et al. [33] investigated 10 children with a history of dietary chloride deficiency and did not find abnormal levels of intelligence quotient based on developmental and psychological testing. Nevertheless, three children were found to have a behavioral pattern that might interfere with school performance. Chutorian et al. [34] investigated 22 children after dietary chloride depletion in infancy and found some cognitive deficits. Willoughby et al. [35] investigated 20 children with a history of symptomatic chloride depletion during infancy. This author found a clear-cut dose–response relationship between the use of a chloride-deficient formula without additional nutritional supplementation and deficient cognitive development at two and four years of age. This author observed a similar tendency in asymptomatic infants exposed to a chloride-deficient formula [36]. Malloy et al. [37] investigated 171 children who had been exposed to a chloride-deficient formula, but without any laboratory work-up. In these children, cognitive development was normal. Malloy et al. [38] compared 35 children of 9–10 years of age in whom hypochloremic metabolic alkalosis developed in association with exposure to a chloride-deficient formula, with a group of 32 similarly exposed children in whom the syndrome was not reported. The results suggested no differences in cognitive, speech, or language development between the two groups. Kaleitha et al. [39] investigated a sample of 13 children with a history of growth deceleration, hypokalemia, hypochloremia, and metabolic alkalosis following exposure to a chloride-deficient formula. Four to nine years later, the children scored within the average range for intelligence.

To sum up, these reports do not provide clear-cut evidence of long-term cognitive deficits after the American outbreak of dietary chloride deficiency. No further studies addressed this issue after 1991. The possible existence of cognitive deficits after the Spanish outbreak was never investigated.

### 4.2. Increased Liking for Salty Foods

Interestingly, a preference for salty foods was noted among 169 young adolescents who had been exposed to a chloride-deficient diet during infancy [40].

## 5. Conclusions

Dietary chloride deficiency was initially observed among infants and was subsequently termed infantile chloride deficiency. We argue against the use of this term because many recent cases were observed in adults. Dietary chloride deficiency, which is likely rare [2,3], more frequently affects infants than older children and adults because the salt balance is positive in this age group. Since the presenting features are not distinctive, a high index of suspicion is required, and the diagnosis is often delayed, especially in isolated cases.

In addition to metabolic alkalosis, the biochemical features of dietary chloride deficiency are hypokalemia and abnormalities that predispose a patient to nephrocalcinosis. Potassium, calcium, and phosphate levels were normal in milks with a low chloride content [1]. It is currently assumed that, in dietary chloride deficiency, hypokalemia results from a potassium redistribution from the extracellular to the intracellular compartment, and from an activation of the renin–angiotensin–aldosterone system. On the other hand, the mechanisms underlying the tendency to develop nephrocalcinosis, which include hypercalcemia, hyperphosphatemia, hypercalciuria, and perhaps also hypocitraturia (secondary to hypokalemia), are poorly understood but unsurprising. Indeed, nephrocalcinosis occurs in rats fed a chloride-deficient diet and in the hypercalciuric variants of Bartter syndrome [1,2].

Dietary chloride deficiency may occur epidemically after marketing of liquid nutritional products lacking in chloride [2,3]. It also occurs sporadically in infants fed with unconventional, often homemade, formulas lacking in this anion. It has also been suggested that dietary chloride deficiency might, in exceptional cases, also develop in breastfed infants (the mechanisms responsible for chloride deficiency in these infants have not been elucidated). Hypernatremia is the most common electrolyte disturbance in breastfed newborn infants. This form of hypernatremia develops in babies who receive too little milk, and should be termed neonatal hypernatremic dehydration secondary to lactation failure [38]. Breastmilk sodium level is slightly increased in these cases. However, breastfeeding-associated hypernatremia is not a form of salt poisoning secondary to salt-rich breastmilk [41].

In the 1970s, suggestions were made to reduce the salt content of infant foods as part of an effort to minimize the development of systemic hypertension in adulthood [42]. Accordingly, the baby food industry resolved to remove added salt from some formula milks, thereby causing the outbreaks of chloride deficiency syndrome.

Chloride accounts for two-thirds of all negative charges and for one-third of tonicity in blood. Nonetheless, the amount of attention this ion receives is limited and much less than other routinely measured electrolytes [43]. Interestingly, the search term “hyperchlor[a]emia” in the National Library of Medicine (2 September 2020) generated 117 citations while “hypernatr[a]emia” generated 1045 citations. The present analysis confirms that the dietary intake of chloride, which was previously considered a “mendicant” ion, indeed plays a crucial role in acid–base and salt balance.

## Figures and Tables

**Table 1:** Common causes of metabolic alkalosis (and hypokalemia) associated with normal or elevated blood pressure.

**Conditions presenting with normal blood pressure**	
**- Chloride deficiency**	
Renal chloride losses	Thiazide or loop diuretics*
	Congenital or acquired chloride (and sodium) losing tubular disorders (e.g.: Bartter or Gitelman syndromes)
Excessive sweating	Physical labor
	Hot and humid conditions
	High sweat salt concentration (e.g.: cystic fibrosis)
Gastrointestinal losses	Vomiting*, nasogastric suction
	Congenital chloride diarrhea, Zollinger-Ellison syndrome, villous adenoma, high-volume ileostomy losses
Transient neonatal chloride deficiency secondary maternal chloride deficiency	Maternal eating disorder
	Mother taking thiazide or loop diuretics
	Mother affected by a chloride (and sodium) losing disorder
Poor dietary chloride intake	
**- Alkali intake (e.g.: alkaline diet, baking soda) or administration, milk alkali syndrome, severe potassium depletion**	
**Conditions presenting with arterial hypertension**	
Liddle syndrome, apparent mineralocorticoid excess syndrome	
Primary hyperaldosteronism	
Excess licorice ingestion	

* sometimes surreptitious.

**Table 2:** Findings in infants of both sexes (no exact information on the sex ratio was available) with failure to thrive, constipation, food refusal, muscular weakness, and delayed psychomotor development fed a low-chloride formula milk.

Finding	Approximate Prevalence (%)	Soundness ^◦^
Blood pressure normal (or low normal)	100	high
Blood parameters		
Metabolic alkalosis (HCO_3_^−^ > 26 mmol/L)	100	high
Hypokalemia (<3.5 mmol/L)	90	high
Hypochloremia (<95 mmol/L)	80	high
Hyponatremia (<135 mmol/L)	70	high
Creatinine, urea, uric acid slightly increased	50	moderate
Renin and aldosterone increased	100	high
Urinary parameters		
Low urinary chloride excretion	100	high
Microhematuria	50	high
Potential for nephrocalcinosis ^△^	50	moderate
Absent juxtaglomerular hyperplasia	100	poor

◦ Scientific soundness of findings was classified as follows: poor (findings supported by studies including <30 patients), moderate (results supported by studies including 30–49 patients), high (findings supported by studies including ≥50 patients); ^△^ tendency to hypercalcemia and hyperphosphatemia, hypercalciuria, and hypermagnesuria.
